# Recent Progress in Photoresponsive Room-Temperature Phosphorescent Materials: From Mechanistic Insights to Functional Applications

**DOI:** 10.3390/molecules30204120

**Published:** 2025-10-17

**Authors:** Yeqin Chen, Yu Huang, Zao Zeng, Guiwen Luo

**Affiliations:** 1 College of Chemical and Engineering, Guizhou University of Engineering Science, Bijie 551700, China; zengzao2025@163.com; 2 Key Laboratory of Photochemical Conversion and Optoelectronic Materials, Technical Institute of Physics and Chemistry, Chinese Academy of Sciences, Beijing 100190, China

**Keywords:** room-temperature phosphorescence, photochromism, information encryption, radical ligands

## Abstract

Room-temperature phosphorescence (RTP) materials with photo-responsive properties have attracted increasing attention for applications in smart luminescent switches, optical logic control, and multidimensional information storage. Compared to other external stimuli, light offers the advantages of non-contact control, high spatiotemporal resolution, and excellent programmability, making it an ideal strategy for reversible and dynamic modulation of RTP. This review summarizes recent advances in light-triggered RTP systems coupled with photochromism. From a structural design perspective, we discuss strategies to integrate photochromic and RTP units within a single material system, covering photoisomerizable molecules, metal–organic complexes, organic–inorganic hybrids, and purely organic radicals. These materials demonstrate unique advantages in fields such as information encryption, bioimaging, and light-controlled upconversion. Finally, future design directions and challenges are proposed, aiming toward high-security, long-lifetime, and multi-channel collaborative luminescent systems.

## 1. Introduction

Organic room-temperature phosphorescence (RTP) materials, characterized by long-lived triplet excitons and large Stokes shifts, have garnered widespread attention in diverse applications such as sensing, bioimaging, display technologies, and data encryption [1,2,3]. To achieve efficient RTP, two fundamental photophysical requirements must be satisfied: (i) enhancing the intersystem crossing (ISC) from the lowest excited singlet state (S_1_) to triplet states (Tn), and (ii) suppressing nonradiative decay from the lowest triplet state (T_1_) The former is typically achieved by introducing aromatic carbonyls, heteroatoms (N/O), or heavy atoms to enhance spin-orbit coupling (SOC), while the latter relies on creating rigid microenvironments through crystal engineering, host–guest confinement, polymer embedding, metal–organic frameworks (MOFs), or cocrystal formation to minimize vibrational dissipation and protect against oxygen quenching [4,5,6,7,8].

Beyond static luminescence, dynamic control of RTP through external stimuli such as temperature [9,10,11], pressure [12], humidity, and pH [13] has been explored [14,15]. Among these, light stimulus stands out due to its non-contact nature, high spatial-temporal resolution, and programmability, making it particularly promising for achieving reversible and controllable RTP. However, implementing light-switchable RTP in molecular crystals or densely packed systems remains challenging, as it requires maintaining the delicate balance between singlet–triplet exciton dynamics and effective suppression of nonradiative decay under photoexcitation.

**Scheme 1:**
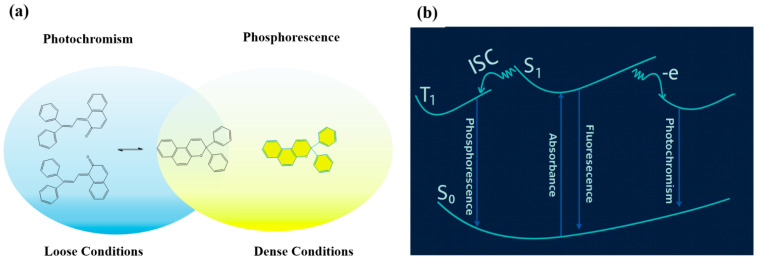
(**a**) Schematic illustration of the intrinsic trade-off between structural rigidity required for RTP and molecular flexibility needed for photochromic transitions. (**b**) Schematic representation of the competitive excited-state relaxation processes between photochromism and phosphorescence.

Photochromism, defined as a reversible photoinitiated change in molecular configuration and electronic structure [16,17,18], offers a potential solution by enabling color and photophysical property modulation [19,20]. The photoinduced configurational changes (such as ring-opening/closing or E/Z isomerization) lead to variations in optical and electronic characteristics, and their reversibility is essential for repeated switching behavior. However, an intrinsic contradiction exists: while RTP requires structural rigidity to stabilize triplet excitons, photochromic transitions typically rely on molecular flexibility. As a concise example, Wang et al.’s [21] naphthopyran-doped polymer shows photochromism without RTP in a loosely cross-linked state, but after curing to a dense network, the RTP appears while isomerization is hindered. This directly illustrated the trade-off: photochromism favors molecular flexibility, whereas RTP demands structural rigidity (Scheme 1a). Meanwhile, the excited-state relaxation pathways of phosphorescence and photochromism are in direct competition. Phosphorescence proceeds via population of the triplet state (T_1_) through intersystem crossing (ISC) from the first singlet excited state (S_1_), followed by radiative decay. By contrast, photochromism commonly originates from photoinduced electron transfer (PET) or related processes that generate (cation) radical species. S_1_ is partitioned between ISC → T_1_ → RTP and PET/isomerization → photochromism, leading to a kinetic trade-off wherein the two channels mutually suppress each other (Scheme 1b).

To address this dilemma, several design strategies have emerged: (i) integrating isomerizable ligands into coordination frameworks or MOFs to couple photochromism and RTP through metal–ligand synergism; (ii) employing host–guest systems with controlled rigidity that preserve molecular freedom necessary for photoisomerization; and (iii) utilizing photoinduced electron transfer (PIET) to generate persistent organic radicals that mediate RTP-photochromism duality.

**Scheme 2:**
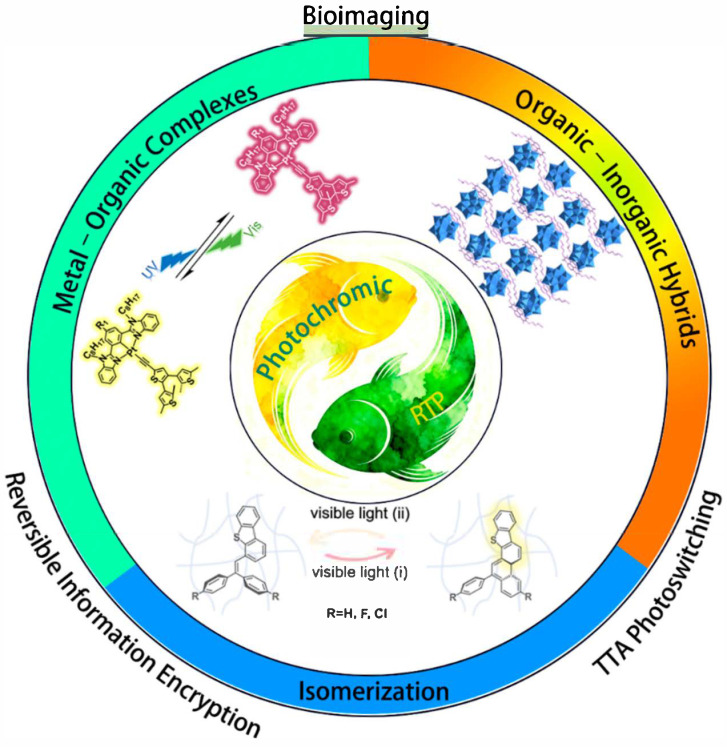
Overview schematic of light-stimulated room-temperature phosphorescence (RTP): mechanisms, molecular design strategies, and representative applications.

This review provides a comprehensive overview of light-responsive RTP materials, with emphasis on molecular design strategies spanning isomerizable ligands, metal–organic complexes, inorganic–organic hybrids, organic radical and covalent–organic frameworks (COFs) systems. It further elucidates the intrinsic correlations among structure, energy levels, and photophysical dynamics, and highlights representative applications in reversible encryption, bioimaging, and multi-channel information storage. Lastly, we discuss current challenges such as fatigue resistance, photostability, quantum efficiency, and visible-light activation, and propose future directions toward rational design of programmable and multimodal light-functional materials (Scheme 2).

## 2. Mechanism of Photoresponsive Room-Temperature Phosphorescent Materials

### 2.1. Isomerization

Certain representative photochromic molecules—such as spiropyrans [22,23,24] (SP), diarylethenes [25,26,27], and naphthopyrans [28]—can undergo reversible ring-opening and ring-closing isomerizations upon UV irradiation or visible light, leading to photoinduced conversion between a less-conjugated open form and a more-conjugated closed form. Taking spiropyran (SP) as an example [29], coordination with Pt(II) ions allows the construction of metal–organic complexes in which SP functions as a photochromic unit, while the heavy-metal center Pt(II), owing to its strong spin-orbit coupling (SOC), facilitates intersystem crossing (ISC) in the organic ligand, thereby enabling room-temperature phosphorescence (RTP) emission.

**Figure 1:**
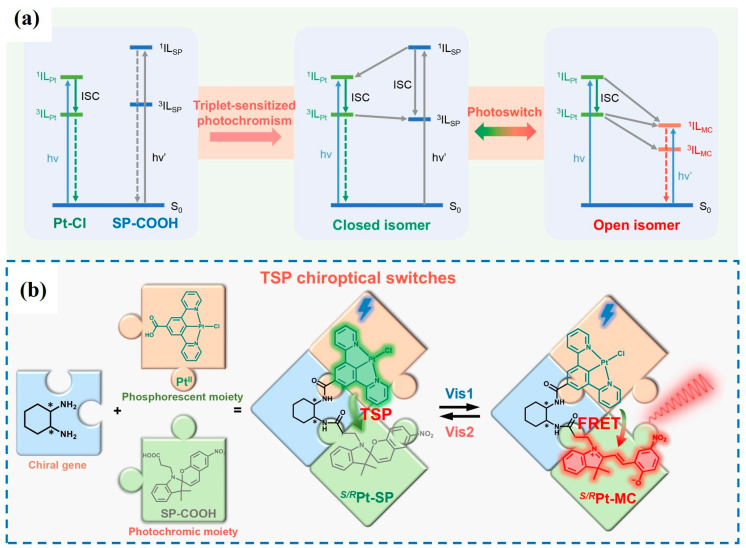
(**a**) Visible-light-controlled molecular switching mechanism of TSP. (**b**) Design strategy for photoreversible CPL switches of S/RPt-SP (λvis_1_ = 420 nm and λvis_2_ = 580 nm) [29]. The asterisk (*) denotes molecular chirality.

During the ring-opening/closing process, the conjugation degree of SP changes significantly, leading to alterations in both the absorption spectrum and electronic energy levels. This structural transformation not only modulates the molecule’s intrinsic absorption properties but also allows reversible tuning of the luminescence pathways of the complex through energy or electron transfer. Consequently, the phosphorescence dominated by the metal-to-ligand charge transfer (^3^MLCT) of Pt(II) can be selectively quenched or enhanced (Figure 1).

This strategy realizes a controllable switch in both RTP intensity and emission characteristics (e.g., color and lifetime) by employing photochromic molecules as the central regulatory element.

#### 2.1.1. Mechanism of Tradition Open-Closed Rings

In recent years, various integrated “photochromism–phosphorescence” systems have emerged by coupling the ring-open/closed isomerization of diarylethene (DAE) or dithienylethene (DTE) with heavy-metal-centered triplet-state emissions. In Ir(III)-centered systems, in 2009, Tian He [30] and co-workers reported the first near-infrared photochromic iridium **complex 1**, where DAE units were directly coordinated to the metal center. Upon 313 nm irradiation, ring-closure occurred; under 550 nm light, the molecule reopened, generating ^3^MLCT-based room-temperature phosphorescence (RTP) at 570 nm in the open form. After ring closure, strong spectral overlap between the new absorption band and the phosphorescence emission leads to internal energy transfer quenching, enabling reversible light-controlled phosphorescence switching. The following year, the same group elucidated the mechanism of **complex 1**: Ir(III) induced strong ISC, and the BTE moiety underwent cyclization along the triplet potential energy surface. In the closed-ring form, the transition character became less metal-centered, resulting in reduced radiative rate (k_r_) and increased non-radiative rate (k_nr_), thus rationalizing the “ring-closed quenching, ring-open emission” programmable switching behavior.

Recently, a series of novel photochromic compounds with RTP was reported, as shown in Figure 1. In 2012, the same group [31] incorporated DTE into a cyclometalated Ir(III) **complex 2**, achieving multistimuli-responsive control via light, chemical, and electrochemical inputs. The DTE unit underwent UV-induced ring closure, sensitized by the ^3^MLCT triplet state through metal-mediated ISC, and reopened under yellow-red light. Additionally, the uncoordinated pyridine sites could be protonated, leading to charge transfer from the metal center to pyridinium, which further quenched emission. In 2016, Cao et al. [32] synthesized two DTE ligands bearing imidazole bridges, **complex 4** and **complex 5**, and their Ir(III) complexes [Ir(dfppy)_2_(tBuL)]·2MeOH and [Ir(dfppy)_2_(tBuLMe)]. While the free ligands showed typical photochromism with emission decline under UV light, their coordination in [Ir(dfppy)_2_(L)] led to energy preferentially channeled into the {Ir(dfppy)_2_} unit, with steric hindrance suppressing ring closure. The system thus retained stable phosphorescent emission and demonstrated a “coordination-suppressed photochromism” contrast.

Subsequently, Cao’s group [33] constructed two Pt(II) cyclometalated complexes [Pt(dfppy)(pbdtmi)]PF_6_ and [Pt(ppy)(pbdtmi)]PF_6_ (ligand dfppy was **complex 7**, ligand ppy was **complex 8**), incorporating DTE-based N^N ligands (**complex 6**). The free ligand exhibited typical DTE-type photochromism (colorless to brown) upon 325 nm irradiation, which was reversible by 600 nm light or in the dark. In the complexes, ring closure was suppressed due to preferential energy transfer to the {Pt(dfppy/ppy)} unit and steric crowding, resulting in weak color change. On the other hand, mechanical grinding of the crystals disrupted dimer formation and “turned off” the emission, while recrystallization with toluene restored the dimer and “turned on” the luminescence.

**Figure 2:**
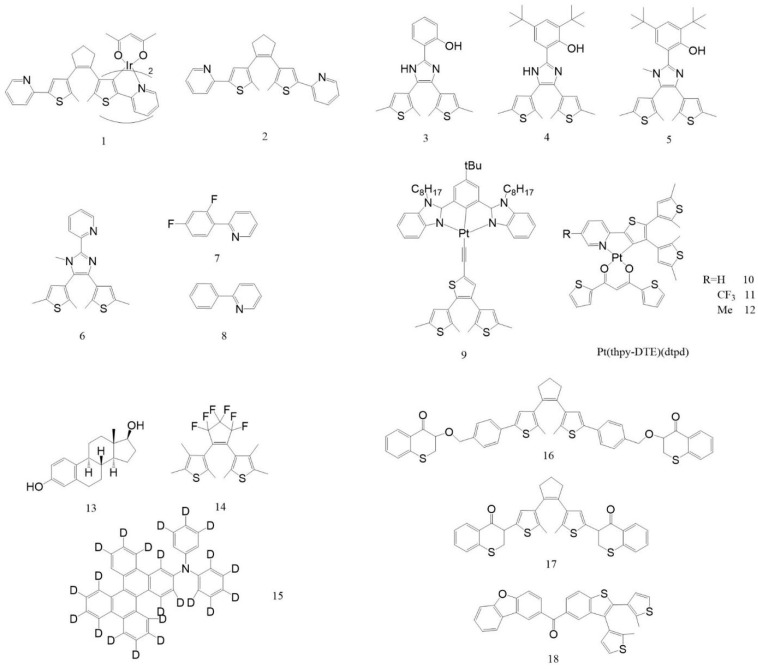
Structures of photochromic diarylethene (DAE) derivatives with RTP effect.

In Pt(II)-based complexes, Yam and co-workers [34] embedded a DTE moiety into the tridentate ligand 1,3-bis(benzimidazol-2′-yl)benzene (bzimb), forming a cyclometalated platinum(II) alkyne **complex 9**, which exhibited both green phosphorescence and reversible photochromism (yellow ⇄ purple) in solution. This behavior originated from light-triggered DTE ring isomerization. Subsequently, Yam et al. [35] designed **complex 10**–**12**, where 420 nm visible light induced DTE-mediated photochromism from yellow to green, accompanied by significant quenching of red phosphorescence originating from a ^3^IL state perturbed by the metal in the open form. Upon ring closure, a new low-energy absorption band emerged (Figure 2).

In purely organic systems, in 2015, Katsurada et al. [36] constructed a ternary photoresponsive RTP platform consisting of an amorphous hydrogen-bonded matrix (**complex 13**), a red RTP emitter (**complex 15**), and a diarylethene-based photochromic molecule (**complex 14**). Within this matrix, **complex 15** exhibits strong red room-temperature phosphorescence (RTP). Notably, the system displays reversible photochromism and emission switching: upon UV irradiation, the diarylethene unit undergoes a ring-closing reaction, generating a new absorption band that overlaps with the excited singlet (S_1_) and triplet (T_1_) states of **complex 15**. This spectral overlap facilitates dual-channel dipole–dipole energy transfer, resulting in substantial RTP quenching. When subsequently irradiated with 540 nm light, the diarylethene reverts to the open-ring form and the RTP is promptly restored, enabling repeatable and reversible emission control.

Building upon this concept, in 2023, Ma Xiang’s group [37] reported a molecular integration strategy of “photochromism × RTP” by covalently linking a highly efficient phosphor, thioxanthone-4-one, with dithienylethene (BTE) through sp^2^/sp^3^ hybridized bonds to obtain **complex 16**/**complex 17**. These molecules exhibit pronounced photochromism in both solution and polymer matrices, and display millisecond-scale RTP when embedded in PVA films. Furthermore, the RTP can be remotely switched ON/OFF by irradiation with different wavelengths.

In 2021, Wang et al. [38] synthesized a single-molecule system **complex 18**, composed of a dibenzofuran phosphorescent unit and a photochromic dithienobenzothiophene (DTBT) moiety. The **complex 18** demonstrates reversible photo-controlled fluorescence/RTP switching in both aggregated states and polymer films. Under 365 nm irradiation, **complex 18** undergoes ring-closure to form c-BFT; upon 580 nm light exposure, it reopens. The new absorption band generated in the closed-ring form overlaps with the RTP emission spectrum, triggering selective phosphorescence quenching via triplet–singlet Förster resonance energy transfer (TS-FRET), while preserving fluorescence. Importantly, this switching process occurs without significant alteration of the crystalline phase. In thin films, visible afterglow ON/OFF switching is clearly observed by the naked eye, and the system demonstrates high fatigue resistance over multiple cycles.

**Figure 3:**
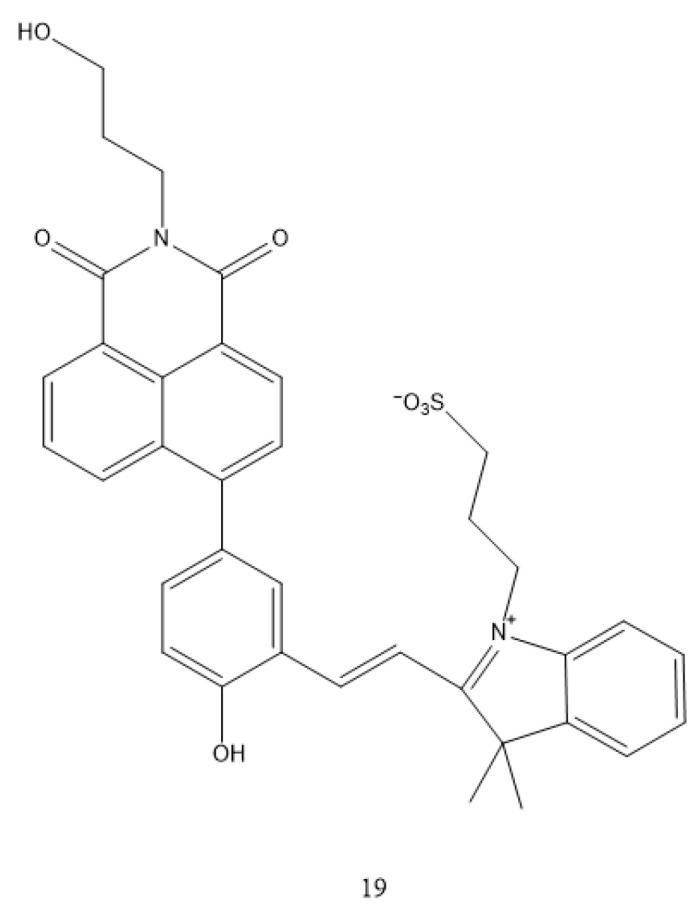
The structure of complex 19.

In addition to the diarylethene (DAE) family, spiropyrans (SPs) represent another important class of photoisomerizable molecules. Building on this, Li Zhen et al. [39] covalently linked a spiropyran unit with a naphthalimide-based RTP emitter to obtain a single-molecule system, **complex 19** (Figure 3). When embedded in a PVP film, **complex 19** undergoes reversible ring-closing/opening transitions under 450 nm blue light, corresponding to an MC-to-SP conversion. This photoisomerization leads to a significant reduction in absorption, a blue-shift in fluorescence, and a pronounced enhancement in RTP intensity and lifetime (from the millisecond to the sub-second range). Furthermore, exposure to ammonia enables the system to extend its emission into the near-infrared region. Remarkably, this system allows for triple-channel optical readout (absorption, fluorescence, and phosphorescence) and temporal decoupling of signals within a single molecular platform, achieving negative photochromism and enabling applications such as remote gas detection and high-density, multichannel optical data storage.

#### 2.1.2. Other Novel Ring-Closing Mechanisms

Room-temperature phosphorescence (RTP) typically requires a rigid environment to suppress non-radiative decay of the triplet state; however, excessive rigidity can hinder the ring-closing reactions of photochromic molecules [21]. Meanwhile, the configurational rearrangements involved in photochromic switching may compete with triplet-state radiative transitions. Consequently, organic molecules relying solely on such configurational isomerization often struggle to achieve both photochromism and RTP simultaneously. To overcome this challenge, a “host–guest and local rigidity” strategy has been proposed, which provides sufficient rigidity to suppress non-radiative losses while maintaining moderate flexibility to accommodate configurational rearrangements, thereby enabling the coexistence of reversible photochromic response and RTP emission [21,38,40,41,42,43].

**Figure 4:**
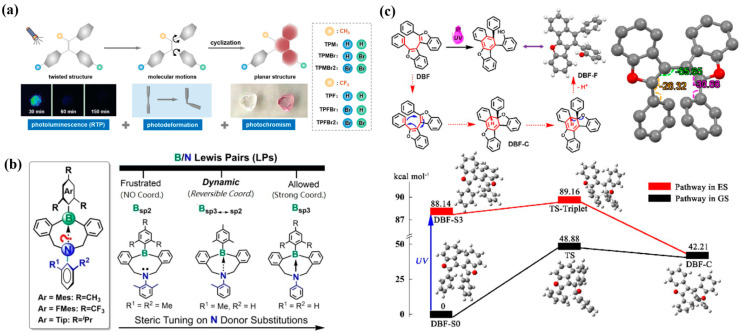
(**a**) Structures and photo-cyclization of triphenylethylene derivatives with multi-photoresponsive behavior [44]. (**b**) Design concept of tunable B/N Lewis pairs via steric/electronic modulation of N → B coordination [45]. (**c**) Proposed photochromism mechanism and crystal structure of DBF-F [46].

In 2022, Li et al. [44] reported a series of triphenylethylene derivatives that realized the integration of “photochromism–photoinduced deformation–RTP.” The underlying molecular process involves UV-triggered photocyclization of the triphenylethylene backbone, leading to extended conjugation and visible coloration (Figure 4a). Bromine substitution reduces the dihedral angle of the photoactive phenyl rings via electron-withdrawing effects, facilitating cyclization, while the heavy-atom effect enhances intersystem crossing (ISC). Newly formed C–H···Br interactions and dense packing in the crystal lattice further rigidify the aggregates and stabilize triplet excitons. Spectroscopically, originally colorless/white samples rapidly turned orange/purple within seconds under 365 nm irradiation, accompanied by new reflection peaks at 480–520 nm. Regarding RTP performance, crystal lifetimes of TPM, TPMBr, and TPMBr_2_ were measured as 13.6, 297.0, and 210.1 ms, respectively, with TPMBr exhibiting an afterglow persisting for 150 min. The system demonstrated reversible light/color/emission modulation within 5 s, with good cycling stability. Furthermore, embedding the molecules into PET films (2.5 wt%) enabled macroscopic bending driven by molecular cyclization, with bending angles up to 130° for TPMBr_2_-PET, indicating fine-tuned control of chromism, deformation, and phosphorescence by irradiation conditions and molecular design.

In the same year, Shi et al. [45] reported a class of dynamic B/N Lewis pair luminophores, among which M1BNM single crystals displayed a coupled “photochromism–phosphorescence activation” effect, achieving an unusual transition from short-lived fluorescence to long-lived RTP (Figure 4b). Upon mild UV or daylight exposure, initially colorless M1BNM crystals rapidly developed a deep violet coloration, which spontaneously faded under continuous irradiation, demonstrating reversible cycling. This unique behavior arose from reversible N → B coordination in dynamic Lewis pairs, while strongly bound or “frustrated” analogs were unresponsive. Spectroscopically, before coloration, the sample exhibited only fluorescence at 495 nm, whereas after coloration, a new phosphorescence band at 535 nm appeared, with green afterglow persisting for 1.3 s. The RTP intensity and lifetime decreased with increasing temperature (2.3 s at 77 K → 84.3 ms at 328 K), consistent with thermal quenching. This enabled reversible light/color/emission control, with cycling between colorless/deep-violet states and fluorescence/phosphorescence emission. Notably, X-ray diffraction revealed negligible structural change before and after irradiation, suggesting that the phosphorescence activation arises from photoinduced electronic/excited-state changes rather than phase transitions, though the precise mechanism remains under investigation.

In 2025, Chen et al. [46] introduced 2,2′-diphenyl-3,3′-dibenzofuran (DBF) as a guest doped into PET, PS, and PVC matrices, achieving for the first time large-area ultrathin films that simultaneously exhibit “photochromism–RTP” coexistence and cooperative regulation (Figure 4c). Industrial-scale films were prepared. UV light triggered a 6π conrotatory photocyclization of DBF, yielding a colored intermediate (DBF-C) responsible for red coloration. Under suitable conditions, DBF-C further underwent C–O bond cleavage to form DBF-F, which preserved phosphorescence but lost chromism. The reversible decoloration process was achieved via white-light or thermal treatment. Spectroscopically, DBF/PET films turned from colorless to deep red within 2–10 s under 365 nm irradiation, accompanied by a new broad absorption band at 525 nm. Delayed emission at 556 nm with lifetimes of 113–185 ms and visible afterglow for 3–4 s was observed across DBF/PET, DBF/PS, and DBF/PVC films, consistent with DBF-originated RTP. The system demonstrated stable chromism and RTP cycling, with enhanced thermal adaptability. Comparison with other hosts highlighted the critical balance between rigidity and flexibility: PVA/PVP matrices showed only RTP without chromism, while PP/PE matrices allowed chromism but no RTP, whereas PET/PS/PVC matrices successfully combined both.

**Figure 5:**
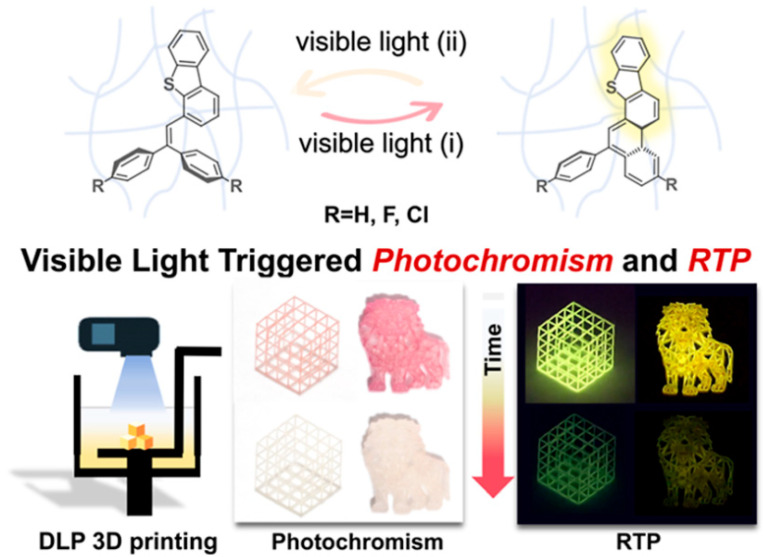
Design of triphenylethylene materials DBTDpH, DBTDpF, and DBTDpCl, and the various printed complex 3D structures [47].

In 2025, HuangWei et al. [47] reported a family of triphenylethylene derivatives (DBTDpH, DBTDpF, DBTDpCl) that exhibit simultaneous photochromism and room-temperature phosphorescence (RTP) under visible light (405 nm) and are compatible with DLP 3D printing (Figure 5). Upon irradiation, the triphenylethylene core undergoes reversible ring-closure/opening photoisomerization, producing a color change from colorless to red. RTP is enabled and stabilized by a host–guest plus local-rigidity design in combination with halogen (F/Cl) substitution, which introduces multiple weak interactions (C–H···π, halogen bonding, etc.) to suppress nonradiative decay and stabilize triplet states, while the polymer matrix selectively restricts nonradiative pathways yet preserves the structural freedom required for cyclization. In the crystalline state, dense C–H···π/C–H···S contacts and edge-to-face π interactions hinder cyclization, so coloration is suppressed. In EA/AA resin films, DBTDpCl shows yellow RTP under 405 nm excitation while the ring-closed isomer appears red, enabling reversible optical control: 405 nm turns on red coloration with yellow RTP; recovery under white/indoor light occurs in ≈12 min, and an afterglow persists for 1.0 s after the light is switched off. The films exhibit excellent cycling stability. Concentration gating (0.01 vs. 1 wt%) allows, within a single 3D-printed object, RTP-only emission or combined coloration + RTP. Theoretical analysis indicates that DBTDpCl dimers possess denser S to T transition channels and enhanced spin-orbit coupling, supporting efficient visible-light-activated RTP and mechanistically rationalizing the coupling between ring-closure photoisomerization and phosphorescence.

### 2.2. Organic Radicals

**Figure 6:**
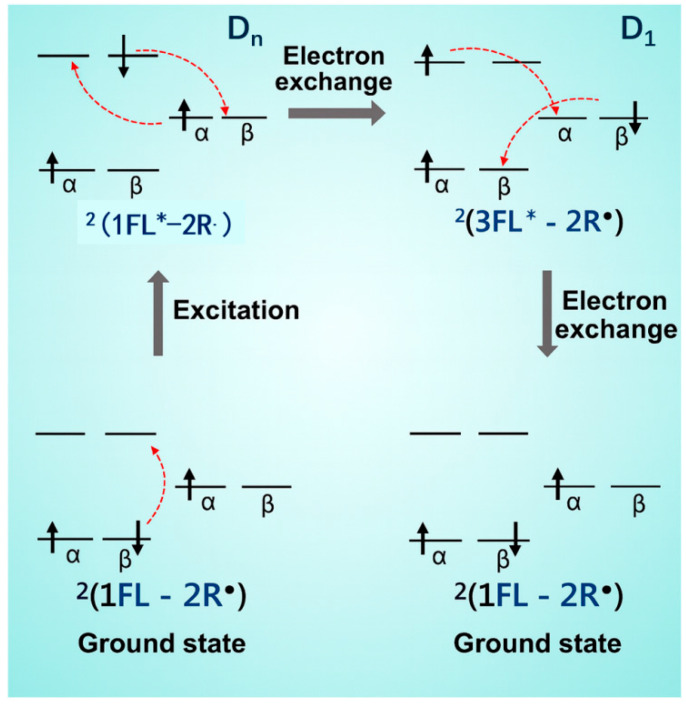
Representative electron-exchange mechanism between fluorophore (FL) and radical (R•) units. (D_n_: singlet excited state coupled to the doublet radical, generating a doublet configuration. D_1_: triplet coupled to a radical doublet).

Organic radicals were initially regarded as non-emissive species and were widely employed as fluorescence quenchers [48]. Their quenching behavior mainly arises from (i) electron transfer, (ii) Förster (dipole–dipole) and/or Dexter (electron-exchange) energy transfer, (iii) electronexchange-induced enhanced ISC, and (iv) enhanced internal conversion (Figure 6) [49,50,51]. Among these, spin-allowed electron exchange is particularly prevalent, converting the excitons of fluorophores into overall doublet states that undergo rapid non-radiative relaxation. In the late twentieth century, researchers discovered that organic radicals could exhibit notable luminescence in the solid state or under rigid microenvironments. Nevertheless, their intrinsic chemical instability and weak emission remained major challenges. Strategies such as introducing steric hindrance and spin delocalization (e.g., polychlorinated triarylmethyl frameworks [52,53]), as well as increasing environmental rigidity, have been shown to improve both their stability and quantum yield.

On the other hand, metal coordination can reshape the excited-state dynamics of closed-shell ligands. Metal–ligand electronic coupling can introduce charge-transfer transitions, while spin-orbit coupling (SOC) can accelerate ISC, thereby enhancing phosphorescence. Consequently, employing organic radicals as ligands to coordinate with metal ions allows precise control of emission color and efficiency while also improving their structural and photochemical stability, thus enabling persistent luminescence under ambient conditions. Importantly, the formation of organic radicals is often accompanied by visible color changes, which confer distinct photochromic effects. This process not only modifies the electronic structure and absorption properties of the molecules but also significantly influences their emission behaviors. This section focuses on the photochromic and luminescent properties of organic radicals, including metal–organic complexes (MOCs), organic–inorganic hybrids, and radical-based small molecules [39,54,55,56,57].

#### 2.2.1. Metal–Organic Complexes (MOCs)

Metal–organic complexes, owing to their structural modularity, cooperative functionalities, and multiple non-covalent interactions, provide an ideal platform for constructing systems with reversible photochromism and emission regulation [58,59]. Within MOCs, the different components play the following roles:

1. Metal ions act as energy donors/acceptors, participate in metal-to-ligand or ligand-to-metal charge-transfer processes (MLCT/LMCT), and enhance SOC to accelerate ISC [60], thereby improving RTP efficiency. Additionally, metal ions serve as nodes to build supramolecular frameworks that rigidify ligand conformations and stabilize excited states [61].
2. Organic ligands bearing electron donor/acceptor groups undergo photoinduced electron transfer (PIET) to generate radicals [62,63,64,65,66]. Radical formation induces distinct color changes and significantly alters emission behaviors, often leading to exciton transfer or quenching. These processes regulate fluorescence or phosphorescence intensity and wavelength, and the reversible radical generation can be controlled by light or heat, typically validated by electron paramagnetic resonance (EPR). Such radical-mediated systems are particularly promising for light-controlled encryption and signal modulation.
3. Guest molecules and weak interactions (hydrogen bonding, π–π stacking, halogen bonding, etc.) fine-tune donor–acceptor distances and orientations, modulating electron transfer efficiency, packing conformations, and consequently, both photochromic rates and RTP lifetimes.

Based on these design principles, researchers have successfully developed various electron-transfer-driven photochromic MOCs that enable reversible tuning of RTP emissions [67]. Upon light irradiation, radical generation induces structural, charge-distribution, or energy-level changes, thereby synchronously altering color and luminescence. Through rational selection of metal ions, donor–acceptor ligands, photochromic units, and RTP-active groups, hybrid crystalline materials with programmable photoresponses have been constructed, offering great promise for multi-information anticounterfeiting, optical sensing, and erasable data storage.

Viologen, as a typical class of electron-accepting molecules, has garnered widespread attention due to its excellent photochromic properties, strong electron-accepting ability, and redox activity. These compounds can undergo single-electron reduction to form deeply colored radical species, making them highly valuable in various applications, including charge-transfer (CT) complexes, redox mediators, molecular electronics, and electrochromic materials. Owing to their unique cationic structures, viologens are often employed as cationic templates or guest molecules, capable of interacting with metal-radical ligands in coordination polymers to regulate the structural and functional properties of the resulting materials [68].

**Figure 7:**
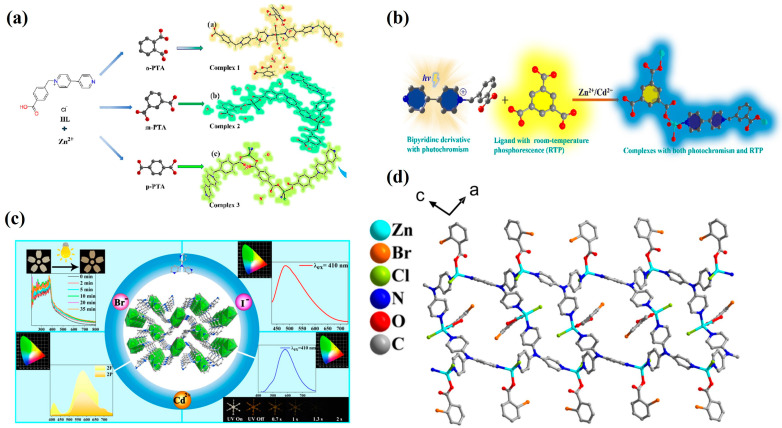
(**a**) Coordination structure of Zn(II) in {[ZnL_2_(H_2_O)_4_]·(oPTA)·(o-H_2_PTA)·nH_2_O} [69]. (**b**) Synthetic strategy of {[Zn(bcbpy)_0_._5_(IPA)]·CH_3_CN} [70]. (**c**) Schematic of [CdX_2_(TPA)] structure with RTP emission [72]. (**d**) Molecular packing of the zinc–organic hybrid [73].

In 2023, Zheng’s group [69] reported a class of coordination compounds constructed from viologen ligands (4-carboxybenzyl-4,4′-bipyridinium chloride), Zn^2+^ ions, and benzoic acid derivatives (o-, m-, p-phthalic acid) (Figure 7a). These complexes exhibited photochromism arising from photo-induced electron transfer (PIET) and radical formation of viologen under light irradiation. During the photochromic process, the materials also displayed tunable long-persistent room-temperature phosphorescence (RTP), with an initial green afterglow emission centered around 520 nm (lifetime on the order of seconds). Upon coloration, enhanced absorption in the 450–550 nm range was observed, accompanied by changes in RTP intensity and slight red-shift. The materials demonstrated excellent reversibility, fully recovering to the initial state upon heating or dark storage.

In 2022, Zheng’s group [70] further developed two viologen-containing coordination polymers, {[M(cbbpy)(HBTC)(H_2_O)]·2H_2_O}ₙ (M = Zn or Cd), that exhibited a triple-response behavior-photochromism, fluorescence, and RTP-within a single solid-state system (Figure 7b). Under 365 nm UV or X-ray irradiation, carboxylic acid-to-viologen PIET generated stable viologen radicals, manifesting as a rapid color change from colorless to navy blue/blue within 10 s. Moreover, the Zn-based system could undergo thermochromism upon dehydration at 120 °C and return to its original state at 180 °C. In the same year, Yang et al. [58] reported a two-dimensional Zn(II)-viologen coordination polymer and its Eu^3+^-postmodified analog (Eu^3+^@Zn-MOF), which exhibited fast, reversible, and multi-stimuli responses (365 nm UV, voltage, ethylamine vapor, and X-ray), with dual fluorescence and RTP emissions.

In 2022, Zheng et al. [59] introduced a one-dimensional coordination polymer composed of 4,4′-bipyridinium derivatives (H_2_bybpy) and CdCl_2_ that featured both photochromic and RTP characteristics. In 2024 [68], two Zn/Cd-based coordination polymers assembled from H_2_bybpy and H_3_BTC were reported, which integrated reversible photo-/electrochromism and persistent low-temperature phosphorescence. Upon 365 nm irradiation, these materials underwent electron transfer and coloration: compounds **1** and **2** changed from colorless to dark blue/navy blue within 120 s and 180 s, respectively, without spontaneous fading over two months at room temperature; full decoloration could be achieved by heating at 100 °C for 1 h.

Beyond viologen systems, similar “photochromism with RTP” phenomena have been demonstrated in various metal–organic materials (including hybrid coordination polymers and MOFs), where the core mechanism generally involves PIET, ligand-to-ligand charge transfer (LLCT), or electron transfer (ET) processes occurring within rigid frameworks, leading to the generation and stabilization of radical species. This enables dual-channel reversible or programmable control over both color and emission. In 2020, Wei et al. [71] employed [(N-MeIQL)BiCl_4_] to demonstrate inorganic-to-organic ET forming N-MeIQL^•^ radicals, leading to concurrent coloration and fluorescence/RTP enhancement, a rare case of “coloration-brightening” synergy. In 2021, Zheng et al. [42] developed a triphenylamine-quinazoline Zn complex that exhibited multi-stimuli white-light/chromatic switching via photo-/thermo-driven ring-opening tautomerism and ESIPT dual-channel emission. In 2022, Xue [62] reported a Dy–phosphate supramolecular system where skeleton-to-protonated pyridylamine ET generated H_3_TPA• radicals, amplifying millisecond-level RTP upon coloration. In 2021, Yan Dongpeng [61] realized RTP on/off switching in isostructural M-(HCOO)_2_(4,4′-bipy) (M = Zn/Cd) systems via LLCT-induced pyridyl radicals, with further expansion to spatially resolved erase–write applications in core–shell MOFs. In two Zn–phosphate/TPB coordination polymers, Feng triggered TPB• formation through HEDP → TPB ET; RTP could be selectively switched on/off based on excitation wavelength and self-absorption competition, enabling erasable patterns and multistep encryption. Subsequently, Yan Dongpeng [64] reported Zn(9-AC)_2_(BIm)_2_ complex showed switchable behavior between crystalline photodimerization (decoloration) and amorphous radical-induced (coloration) channels, enabling bidirectional photochromism and emission modulation. In 2024, Xue [72] constructed [CdX_2_(TPA)] (X = Br/I), where halide-to-TPA PIET generated radicals and cooperated with RTP; emission intensity decreased continuously with coloration while maintaining structural stability (Figure 7c). In same year, Wang [73] designed [Zn_3_TPA_2_(2-BrBA)_3_Cl_3_], which exhibited dual photo-/mechanical responsiveness via 2-BrBA → TPA ET. During coloration, combined self-absorption suppressed both fluorescence and RTP, whereas grinding-induced amorphization reversibly enhanced RTP (Figure 7d). These studies collectively demonstrate that precise design of donor–acceptor pathways and local rigidity within coordination/supramolecular frameworks can enable integrated regulation over multiple optical parameters—including on/off switching of coloration and phosphorescence, lifetime, chromaticity, and even chirality—while preserving structural robustness and cycling performance. Such systems hold promising potential for advanced applications in information encryption, anti-counterfeiting labels, rewritable printing, and spatiotemporally resolved optoelectronic devices.

#### 2.2.2. Organic–Inorganic Hybrids

In organic–inorganic hybrid photochromic systems, photoinduced valence state modulation serves as one of the key mechanisms triggering reversible color changes. Typical transition metal oxides such as WO_3_ [74], MoO_3_ [75,76], V_2_O_5_ [77], TiO_2_ [78] can undergo valence transformations related to electron injection or extraction under UV or visible light irradiation, resulting in pronounced and reversible color-switching behavior [79,80,81,82,83,84,85,86,87,88,89,90,91]. To integrate optical functionality with processability, recent efforts have focused on constructing multi-level organic–inorganic hybrid materials by coupling organic photochromic units with polyoxometalates (POMs). Through supramolecular interactions such as hydrogen bonding, metal coordination, π–π stacking, and anion–π interactions, the organic and inorganic components are assembled into synergistic architectures that enhance response speed, durability, and film-forming capability [92,93,94,95]. More importantly, combining electron-deficient π-systems with electron-rich POM anions allows for photoinduced electron transfer (PIET) between organic chromophores and POM clusters, leading to the generation of stable radical species.

**Figure 8:**
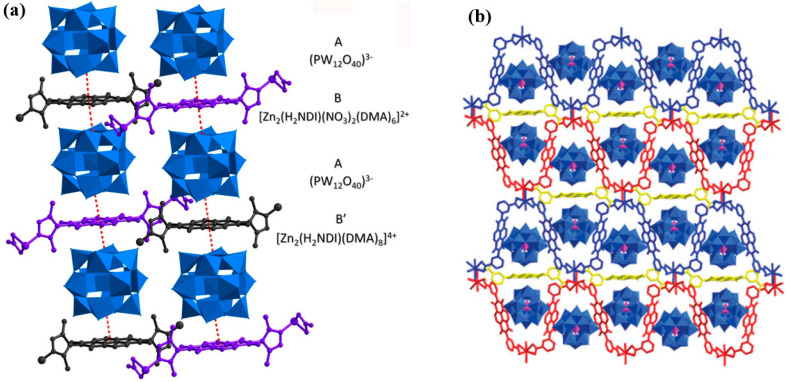
(**a**) ABAB′ packing induced by anion–π interactions (red dashed lines) [96]. (**b**) Crystal framework of 1-Lu viewed along the a axis [97].

In 2018, Lu [96] et al. reported the first example of an organic–inorganic hybrid system featuring charge transfer (CT) between POMs (PW_12_O_40_^3−^) and naphthalenediimide (NDI) units. In the crystalline structure, CT pathways were established via anion–π and C–H···anion interactions across spatial domains (Figure 8a). Upon exposure to blue light (460–465 nm) or 350 nm UV, electrons were transferred from the POM to the NDI, generating NDI radical anions along with valence changes in tungsten centers. This process resulted in macroscopically visible coloration, which could spontaneously fade in the dark, and PXRD/IR analyses confirmed that the overall framework remained structurally intact. Photophysically, the system exhibited red room-temperature phosphorescence (RTP), whose intensity gradually decreased upon continuous irradiation. This was attributed to the modulation of the ICT bridging state, which slightly reduced intersystem crossing (ISC) efficiency. Consequently, reversible regulation of light/color/emission was achieved through “photoinduced coloration–dark decoloration” gating, enabling modulation of metal-free NDI-based local RTP by tuning CT states while preserving lattice stability. This work highlights the potential of unconventional anion–π interactions in constructing spatially bridged CT pathways that endow POM-based hybrids with dual photochromic and phosphorescent functionalities.

In 2020, Lin et al. [60] developed a series of organic–inorganic hybrid crystals based on self-assembly between dipyridyl-terminated naphthalenediimide (DPyNDI) ligands and POM clusters via hydrogen bonding and electrostatic interactions. These materials exhibited fast and reversible photochromism in the solid state. Under visible light irradiation, the crystal color gradually changed from pale to dark due to photoinduced electron transfer from the POM to the DPyNDI moiety, generating NDI radical anions (NDI•^−^) and reduced “heteropoly blue” POM species. This process was reversible in the dark or could be manipulated via controlled light exposure. More significantly, these hybrid crystals also enabled switchable RTP under light irradiation. Before exposure, the materials exhibited short-lived fluorescence mainly originating from ligand-centered emission. Upon light activation, the formation of radical species led to intramolecular charge transfer (ICT) states, which enhanced intersystem crossing (ISC) efficiency. Under the combined effects of the rigid crystalline lattice and strong anion–π interactions, long-lived RTP emission emerged. The phosphorescence quantum yield correlated positively with the strength of anion–π interactions, demonstrating the structure–property relationship.

In 2020, Lu et al. [97] reported a host–guest solid-state material constructed from Keggin-type polyoxometalates (POMs) and rare-earth organic layers containing naphthalenediimide (NDI) ligands (Figure 8b). The material exhibited both strong red room-temperature phosphorescence (RTP) and reversible photochromism, owing to through-space charge transfer (CT) pathways established via anion–π interactions between POM and NDI units. Under simulated sunlight irradiation for 30 min, the sample changed color from pale yellow to light brown, which spontaneously faded in the dark. All 1-Ln complexes emitted red RTP under 410 nm excitation, and continuous irradiation led to a decrease in RTP intensity to approximately 12% of its initial value. The mechanism of reversible “light/color/emission” modulation was attributed to donor–acceptor CT channels facilitated by tight anion–π stacking. This configuration not only reduced ΔE_ST_ and enhanced intersystem crossing (ISC) to generate RTP, but also induced significant spectral overlap between the photo-generated W(V) absorption band and NDI emission, triggering a FRET-type quenching pathway. Upon dark storage, the W(V) species were oxidized, restoring the CT state and gradually recovering both the color and emission properties, thereby realizing a switchable coupling of color and phosphorescence.

**Figure 9:**
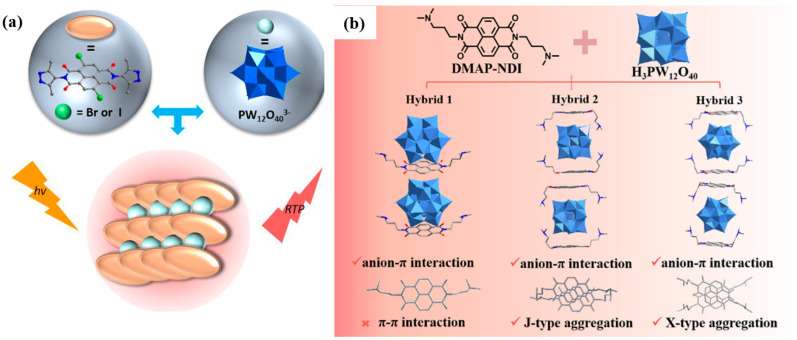
(**a**) Design strategy of Br or I core-substituted NDI-POM D-A hybrid crystals for RTP emissions [98]. (**b**) Structural differences in NDI–POM donor–acceptor hybrids [99].

In 2021, Lin et al. [98] reported two donor–acceptor (D–A) hybrid crystals (denoted as 1 and 2) self-assembled from phosphotungstic acid anions (PW_12_O_40_^3−^, donor) and halogenated NDIs (Br_2_NDI and I_2_NDI, acceptors), via weak anion–π and other supramolecular interactions (Figure 9a). These systems exhibited coupled photochromism and RTP, regulated via light-driven electron transfer. Upon photoexcitation, electrons were transferred from POM/NMP to NDI, forming NDI radical anions (NDI•^−^) and reduced W(V) species. Macroscopically, crystal 1 changed from yellow to near-black under light and spontaneously decolored in the dark; 2 exhibited radical generation and coloration even under ambient light. Both materials emitted red RTP, whose intensity gradually diminished upon several minutes of continuous irradiation, yet remained spectrally unchanged and recoverable in the dark. This enabled reversible light/color/emission control, where light-induced coloration and dark recovery acted as gating mechanisms. The photoinduced W(V) absorption band overlapped with the NDI emission band, resulting in self-absorption-type quenching and weakened excitation efficiency, dynamically modulating RTP intensity and lifetime. Simultaneously, the presence of heavy atoms and optimized energy alignment enhanced ISC, yielding stronger RTP in Br_2_NDI, whereas I_2_NDI showed greater color sensitivity.

Subsequently, Lin et al. [99] used NDI–POM donor–acceptor hybrids as a model system to systematically elucidate the role of anion–π versus anion–π–π interactions in regulating coupled photochromism and RTP (Figure 9b). In three crystalline systems (1–3), system 1 contained only anion–π interactions, 2 introduced additional π–π stacking (forming anion–π–π motifs), and 3 possessed overly strong interactions leading to instability and emission quenching. Among them, 1 exhibited the fastest photo-response and highest RTP quantum yield; 2 showed slower and weaker emission due to exciton localization and non-radiative dissipation induced by π–π stacking; 3 was unstable and non-emissive. The observed molecular behavior was governed by photoinduced electron transfer (PIET): under xenon lamp irradiation, electrons transferred from POM to NDI, forming NDI•^−^/W(V) pairs and resulting in color change. In terms of reversible “light/color/emission” modulation, the materials underwent multiple cycles of photochromism and recovery. During coloration, the newly formed absorption bands overlapped with excitation, leading to emission quenching (more significant in 2). By weakening π–π stacking and strengthening anion–π interactions, the design could be tuned toward faster response and higher RTP output.

#### 2.2.3. Radical-Based Small Molecules

**Figure 10:**
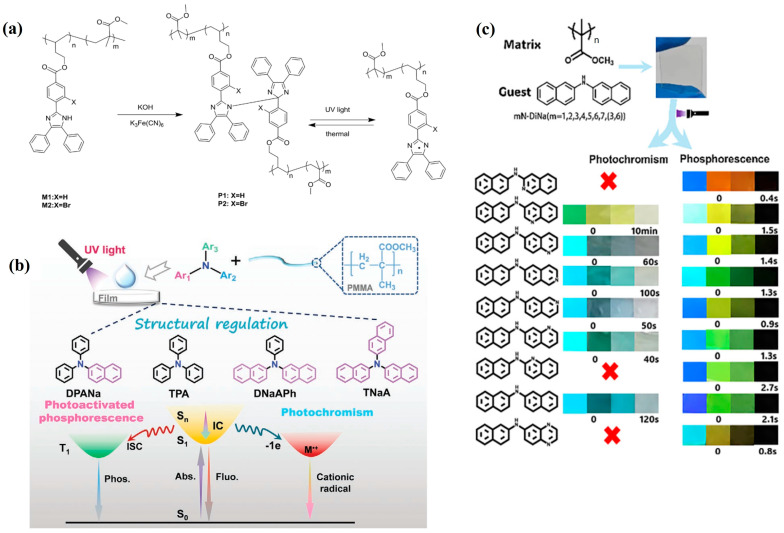
(**a**) General synthesis of HABI [100]. (**b**) Photoactivated RTP and photochromism of triarylamines in PMMA [101] (**c**) Doping strategy and dual-responsive behaviors of nN-DiNa/PMMA films [102].

Focusing on the synergistic regulation of “radicals–energy levels–microenvironments”, recent studies have progressively achieved programmable coupling of photochromism and room-temperature phosphorescence (RTP) within unified material systems. In 2021, Ma et al. [100] employed reversible bond cleavage/reformation of HABI moieties embedded in rigid cross-linked polymers (Figure 10a). Upon light irradiation, triphenylimidazole radicals are generated, whose new absorption bands overlap with the phosphorescence emission, enabling synchronous modulation of RTP intensity along with the yellow-to-brown photochromic transition. Moreover, the system supports stable write–erase cycling via UV writing and thermal erasure.

As a complementary strategy, in 2020, Bo et al. [41] introduced norbornene-fused aromatic frameworks to reduce π–π stacking distances and enhance intermolecular interactions. This simultaneously promoted ISC and suppressed nonradiative decay of triplet excitons, resulting in significantly enhanced afterglow in purely organic crystals, along with unexpected long-retention solid-state photochromism. This allowed for multi-channel information encoding based on spatial, temporal, and luminous contrast.

Further extending this concept, in 2024, Cheng et al. [55] developed helical nanofibers by co-assembling chiral dopants and copolymers in liquid crystalline matrices. Blue/UV light and photothermal activation triggered the formation of stable radical pairs, which not only amplified triplet emission but also transferred chirality from inducers to emissive units, enabling cooperative modulation of color and circularly polarized RTP (CP-RTP). The system thus supported multi-parameter programmable switching.

On another front, in 2021, Li et al. [101] explored homologous tris(arylamine) derivatives embedded in high-Tg matrices, revealing an alternative pathway based on homolog differentiation (Figure 10b). Certain molecules underwent photoinduced electron transfer to form colored radical cations, while others consumed oxygen under illumination, thereby suppressing triplet quenching and activating RTP. This enabled environmentally gated, reversible switching of both color and phosphorescence on a single material platform.

Following a site-engineering strategy, in 2025, Ma et al. [102] programmably introduced nitrogen-containing centers into naphthalenamine skeletons, enabling UV-induced generation/reversion of radical cations and thus reversible color change between colorless and colored states (Figure 10c). By modulating the energy gaps of neutral and radical species and tuning ΔE_ST_/ISC, effective coupling between “photochromism” and “phosphorescence” channels was achieved, resulting in color-tunable ultralong RTP. The material demonstrated robust multi-cycle write–erase capability in both films and bulk solids.

In 2024, Yan Dongpeng et al. [103] developed a zero-dimensional (0D) halide hybrid crystal (DBMI–TeA) that exhibits photochromism and room-temperature phosphorescence (RTP) duality. The system leverages the formation of organic radicals upon UV irradiation, leading to a color change from yellow to dark brown alongside the emergence of a bright yellow phosphorescent emission centered at 570 nm. The crystal structure features isolated DBMI^2−^ and TeA^+^ units, where spatial confinement suppresses nonradiative decay, facilitating ultralong RTP. Importantly, upon halting irradiation, both the coloration and RTP vanish rapidly, allowing reversible switching. The system also demonstrates excellent photofatigue resistance, maintaining performance over 50+ write–erase cycles.

Overall, strategies have evolved from stabilizing radicals in polymers and leveraging spectral overlap to control emission, to enhancing triplet states through rigid π-fusion and dense packing, and further to chiral co-assembly for CP-RTP enhancement, homolog-specific behavior, and precision site engineering for stiffness-energy alignment. These advances collectively point to universal design principles for achieving orthogonal or synergistic switching of photochromic RTP, which have shown practical feasibility in rewritable printing, secure anti-counterfeiting, and multidimensional information storage.

### 2.3. COF

In recent years, covalent organic frameworks (COFs)—with programmable organic building blocks and rigid, porous lattices—have emerged as ideal platforms for coupling photochromism with room-temperature phosphorescence (RTP) On the one hand, lattice rigidification and confined microenvironments effectively suppress nonradiative decay, stabilize triplet excitons, and enhance RTP contrast [104,105]; on the other, well-defined channels and tunable coordination/hydrogen-bonding sites provide designable interfaces for conformational/configurational change, host–guest interactions, and energy-level tuning, enabling photochromic processes [39,106,107,108] (e.g., ring opening/closing, E/Z isomerization, PET-mediated switching) to operate synergistically with triplet management in a single framework while retaining excellent photo/thermal stability and cycling durability. Notably, the ordered pores of COFs can host metal sites or guest molecules to create multiple emitting centers/traps; selective excitation of distinct centers, or optimization of host–guest energy transfer pathways, then affords excitation-dependent (Ex-de) luminescence color changes, delivering multicolor, programmable, and long-lived emission/afterglow across the visible–near-UV region.

**Figure 11:**
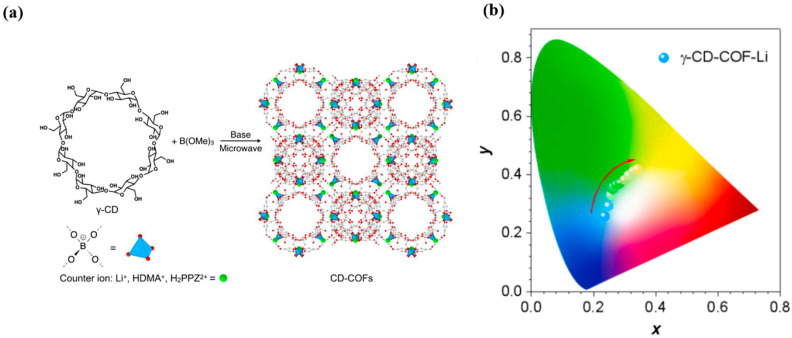
(**a**) The preparation of γ-CD-COF-Li. (**b**) Trajectory of tunable p-RTP colors of γ-CD-COF-Li, recorded by changing the *λ*_ex_ from 245 to 365 nm [109].

Building on this concept, in 2024, Yuan et al. [109] employed cyclodextrin-based systems further integrated with COFs and host–guest inclusion (γ-CD-COF-Li) to realize cluster rigidification via multiple hydrogen bonds/short contacts, achieving blue-to-green tunable long-afterglow RTP under 245–365 nm excitation (Figure 11). In 2025, Nandi et al. [110] coordinated Sm^3+^ to a pyrimidine COF (PyCOFs), coupling rare-earth levels with N-sites in a multi-trap, interlayer-stacked network to produce excitation-redshift–induced emission redshifts. In 2025, Wang et al. [111] covalently embedded COFs-2DPA into a PA6 backbone; the combined “covalent + hydrogen-bonding” rigidification and energy-level reconfiguration yielded concurrently tunable fluorescence and delayed phosphorescence under 365 nm excitation, with lifetime and color programmable via filler content.

In sum, COFs leverage framework rigidification and confinement, and can incorporate metal sites or guest adsorbates to generate multiple emitting centers/traps, thereby enabling programmable, visible-light-tunable emission and long afterglow; their strengths include structural designability, high stability, mechanistic tractability, and extensible form–function integration.

## 3. Applications of Photoresponsive Room-Temperature Phosphorescent Materials

### 3.1. Reversible Information Encryption

The demand for anti-counterfeiting materials has risen sharply in recent years owing to the proliferation of forged and duplicated security documents [28,112,113,114]. It is therefore urgent to design high-security systems featuring distinct colors and multi-modal authentication to enhance document safety and deter counterfeiting. In this context, photoluminescent and photochromic materials have been widely implemented in security inks and secure printing [115,116,117,118,119,120].

**Figure 12:**
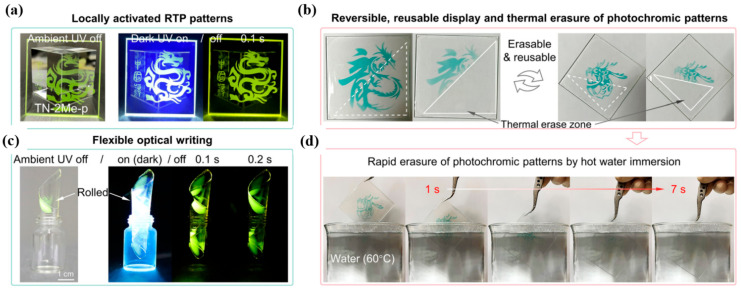
Photo-induced RTP and thermally activated photochromism with dynamic RTP for erasable information encryption [54]: (**a**) Daytime and nighttime afterglow images of TN-2Me-p film drop-coated on a quartz substrate. (**b**) Room-temperature photochromic patterns on TN-2Nap-p film, fading within 20 min and erasable by localized heating (white triangle). (**c**) Photo-induced RTP from a peeled film curled into a 1.0 cm-diameter cylinder. (**d**) Re-exposed “Loong head” pattern erased in 7 s by immersion in 60 °C water.

In 2024, Li et al. [54] reported PMMA films doped with three thianthrene derivatives (TN-2Me/PhNap/2Nap) that exhibit coupled, reversible control of photo-induced RTP and thermally activated photochromism. Under continuous UV irradiation, oxygen is first depleted, amplifying triplet radiative pathways and turning on RTP; concurrently, cation-radical formation occurs in selected derivatives, producing coloration radicals that were directly evidenced by EPR. The radical process is strongly temperature-promoted (e.g., TN-2Nap-p accelerates from hundreds of seconds at room temperature to seconds at 80 °C), whereas at liquid nitrogen temperature, long afterglow appears without coloration, indicating thermally activated chromism. Spectroscopically, RTP lifetimes increase (to 0.13, 0.48, 0.43 s for the three films), and the quantum yield of TN-2Me-p rises markedly; coloration is accompanied by growth of a new 640 nm absorption that tracks color depth, with substituent-dependent radical decay kinetics. These features enable reversible light, color, emission programming (Figure 12): UV through masks writes patterns and enhances afterglow, which fades upon oxygen back-diffusion; photochromic coloration self-erases at ambient conditions or is rapidly erased by heating. The films show minimal fatigue over multiple cycles and support millimeter-scale resolution and flexible deformation, demonstrating potential for information encryption/anticounterfeiting, erasable optical printing, and flexible displays.

**Figure 13:**
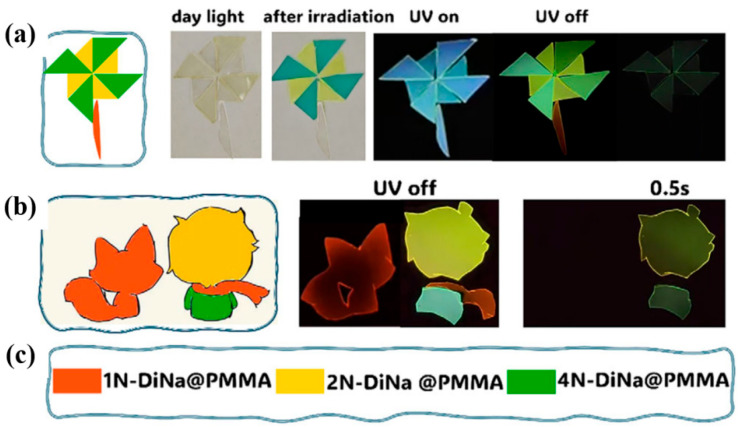
(**a**) Color-changing pinwheel under daylight and under darkness; (**b**) a vivid cartoon of “Boy and Fox”. (**c**) The design concepts for the applications of colored room temperature phosphorescence and photochromism [102].

In 2025, Ma et al. [102] developed a family of nitrogen-substituted dinaphthylamine (DiNa) derivatives (including 1N–7N-DiNa and 3,6N-DiNa) and embedded them in PMMA films to provide moderate rigidity and processability, thereby integrating tunable ultralong room-temperature phosphorescence (UORTP) with reversible photochromism in a single platform. Photoinduced formation of cation radicals-readily verified by EPR-triggers coloration, while the electron density at –NH– sites and the energy gap between the neutral species and the corresponding radicals govern color stability and bleaching kinetics. Concurrently, the UORTP lifetime and intensity are dictated by the intramolecular ΔE_ST_ and intersystem crossing (ISC) efficiency, establishing a controllable coupling between the “chromic channel” (radical-associated absorption/self-absorption) and the “phosphorescent channel” (triplet population/nonradiative suppression) along both energetic and excitonic pathways. Leveraging this construction and mechanism, the authors realized erasable anti-counterfeiting/encryption (Figure 13): short UV photolithography on DiNa@PMMA films writes dual-mode information (color + afterglow, e.g., QR codes and multicolor patterns), which can be erased spontaneously in the dark or accelerated by heating and rewritten over multiple cycles; dual-parameter readout-hue together with phosphorescence lifetime/intensity enables higher-security identity verification and information encryption.

Collectively, these studies demonstrate that coupling photochromism with RTP enables programmable, rewritable, dual-mode authentication (color and lifetime), substantially elevating security and information density. The key control levers are oxygen management/radical thermodynamics. Practical bottlenecks remain—most notably UV-only activation, environmental sensitivity (O_2_/humidity), and radical fatigue.

### 3.2. Bioimaging

**Figure 14:**
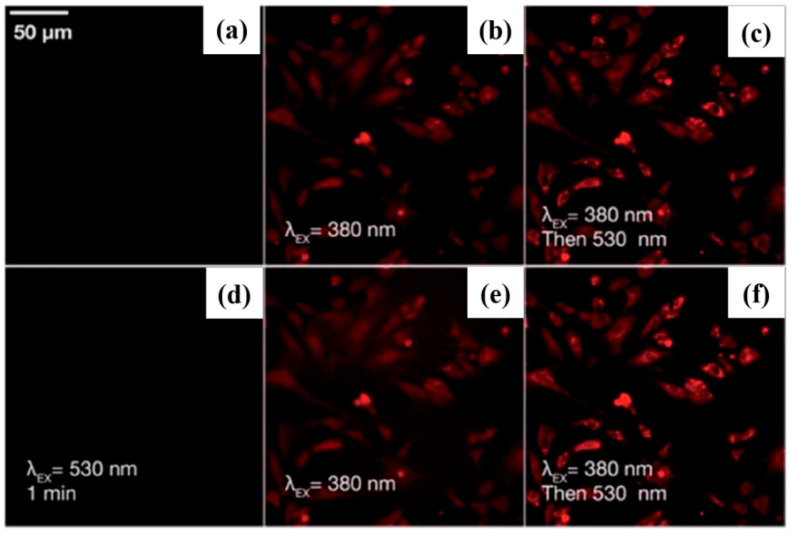
(**a**–**f**) Fluorescence images of 1o-incubated HeLa cells showing light-induced red emission: (**a**) before irradiation, (**b**) after 30 s of 380 nm light, (**c**,**d**) after 530 nm excitation, (**e**) after second 380 nm irradiation, (**f**) after brief 530 nm exposure [121].

In bioimaging, photochromic phosphorescent materials offer programmable color together with long-lived emission, thereby combining spatial–temporal control with time-gated readout. For example, in 2020, in a quinoline-substituted diarylethene (DAE) family, the same molecular scaffold leverages ring-opening/closing isomerization and emission-channel partitioning to achieve synergistic regulation of reversible photochromism and room-temperature phosphorescence (RTP), enabling single-molecule localization–based super-resolution imaging and suggesting promise as small-molecule RTP probes [121] (Figure 14).

**Figure 15:**
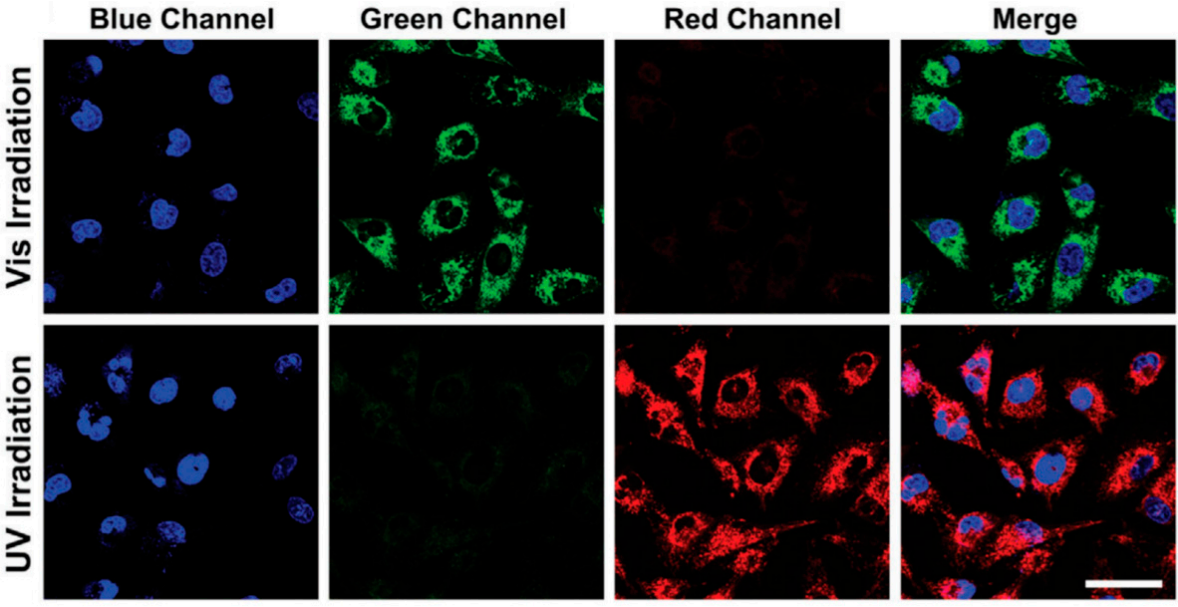
Cellular imaging of A549 cancer cells costained with the G3⊂CB[7]@SC4A4/SP or G3⊂CB[7]@SC4A4/MC assembly. 4,6-Diamidino-2-phenylindole (DAPI, blue) was used to stain the nuclei (scale bar = 30 μm) [122].

In aqueous media, in 2022, Liu et al. [122] cascaded an RTP donor (G3⊂CB\[7]@SC4A4) with a photochromic acceptor, spiropyran (SP), to realize reversible UV/visible switching between donor RTP and merocyanine (MC) delayed fluorescence. This system enabled light-controlled multicolor labeling of A549 live cells with low cytotoxicity, remote addressability, and time-resolved detection, demonstrating the feasibility and safety of programmable emissive platforms for cellular imaging (Figure 15).

**Figure 16:**
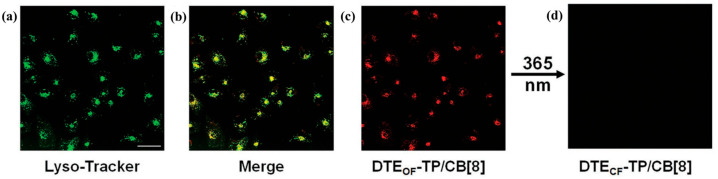
Laser confocal microscopy images of (**a**) Lyso-Tracker Green, (**b**) merged image, and (**c**) DTE-TP/CB[8]. (**d**) Laser confocal images of DTE-TP/CB[8] after 365 nm light irradiation [123].

Targeting near-infrared (NIR) readout, in 2022, Wang et al. [123] constructed a DTE-TP/CB[8] host–guest complex in which CB[8] induces intramolecular folding and amplifies intramolecular charge transfer to turn on NIR-RTP, while the DTE unit undergoes UV/visible-driven ring isomerization to couple chromism with phosphorescence. The complex delivers light-switchable NIR phosphorescence from lysosomes in live cells and, in combination with IR780, extends into longer-wavelength channels, affording high cell viability and suitability for deep-tissue imaging and encrypted tagging (Figure 16). Collectively, rational molecular and supramolecular designs can preserve biocompatibility while affording multi-dimensional tunability (visible/NIR, color/lifetime), providing powerful tools for precise and vivid bioimaging.

Coupling photochromism with RTP delivers programmable, time-gated bioimaging that unites spatial write/erase control with lifetime contrast across the visible–NIR window, while maintaining preliminary biocompatibility via molecular/supramolecular design.

### 3.3. TTA Photoswitch

**Figure 17:**
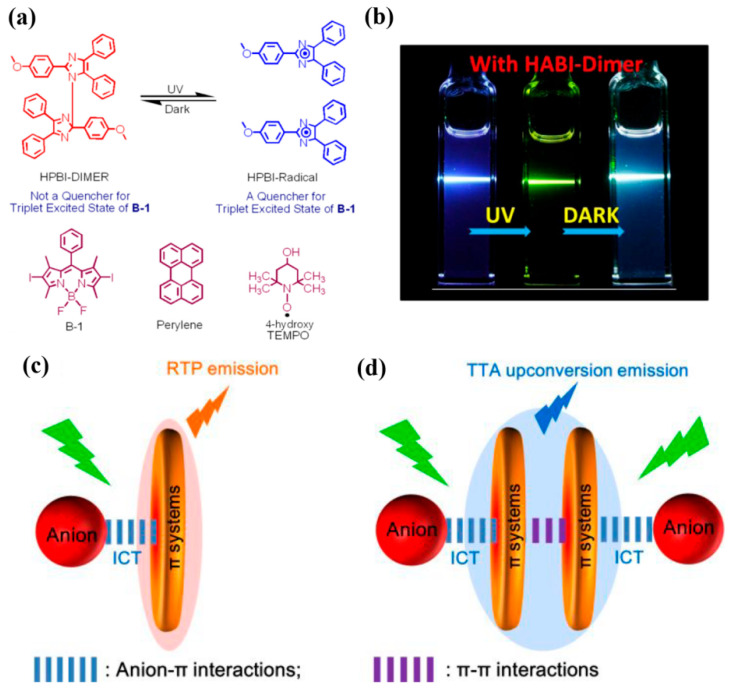
(**a**) Photochromism of HPBI with structures of the triplet sensitizer (B-1) and acceptor (perylene) for TTA upconversion. (**b**) Reversible TTA-UC photoswitching using HABI-dimer under UV/dark cycles [131]. (**c**) Schematic of anion–π interaction-induced RTP. (**d**) Schematic of TTA upconversion emission [132].

Triplet–triplet annihilation upconversion (TTA-UC) proceeds via the fusion of two triplet excitons to generate a higher-energy singlet [124,125,126,127,128]; accordingly, quenching of the acceptor triplet suppresses [129,130] TTA-UC. Leveraging this principle, in 2017, Zhao [131] et al. devised a ternary system comprising a photochromic precursor-hexaphenylbiimidazole (HPBI) dimer-a BODIPY sensitizer (B-1), and a pyrene acceptor. Upon 365 nm irradiation, the HPBI dimer undergoes homolytic cleavage to a persistent organic radical (with emergent 500–700 nm absorption), which efficiently quenches the B-1 T_1_ state via a radical–triplet pair mechanism (RTPM), switching the B-1/pyrene TTA-UC from ON to OFF. In the dark, the radical re-dimerizes to HPBI, restoring the sensitizer triplet population and thus reversibly turning TTA-UC back ON (Figure 17a).

Building on donor–acceptor design, in 2021, Lin [132] et al. constructed four hybrid D–A crystals (1–4) in which a twisted tetrachloro-substituted perylenediimide (4Cl-DPPDI) self-assembles into a one-dimensional racemic hydrogen-bonded network and co-crystallizes with Keggin-type polyoxometalates (XM_12_O_40_^n−^; X = P/Si, M = W/Mo). Anion–π contacts bring POM and 4Cl-DPPDI into close proximity, while lone-pair–π-assisted face-to-face dimerization fixes DPPDI pairs, yielding reversible photochromism and unconventional room-temperature phosphorescence (from an ICT-bridged ligand-localized triplet). In this configuration, adjacent 4Cl-DPPDI triplet acceptors at short separation undergo triplet–triplet fusion, producing a high-energy singlet that emits short-wavelength upconverted fluorescence (440 nm); the upconversion efficiency is governed by the triplet density and the dimer spacing, the latter tuned by the strengths of the anion–π and lone-pair–π interactions. Under continuous visible irradiation, POM → PDI photoinduced electron transfer generates PDI radical anions and reduced POM species, introducing new visible/NIR absorptions and causing time-dependent attenuation of both RTP and TTA emissions with largely unchanged peak positions; partial recovery occurs in the dark, enabling reversible, coupled control of color, emission, and upconversion (Figure 17b).

These studies illustrate two complementary routes to light-programmable TTA upconversion (TTA-UC) coupled with RTP: (i) radical gating of the sensitizer triplet (HPBI↔radical) that toggles TTA-UC ON/OFF via a radical–triplet pair mechanism, and (ii) crystal-engineered donor–acceptor lattices (POM·4Cl-DPPDI) that co-enable reversible photochromism, unconventional RTP, and short-wavelength UC fluorescence through controlled dimer spacing and anion–π/lone-pair–π interactions.

## 4. Discussion: Synthetic Requirements, Advantages, and Limitations Toward Practical Applications

Despite the rapid advances in photochromic room-temperature phosphorescent (RTP) materials, several intrinsic challenges continue to impede their broad practical implementation. A fundamental contradiction persists between the rigid molecular environments required to suppress non-radiative decay and stabilize triplet excitons for RTP, and the structural flexibility essential for efficient photoisomerization. This trade-off hinders the simultaneous optimization of photoresponsiveness and phosphorescent performance.

From a molecular design standpoint, high-performance photochromic–RTP systems demand precise structural engineering. In metal–organic hybrid materials, coordination frameworks must allow for reversible isomerization while maintaining strong spin-orbit coupling (SOC) to facilitate intersystem crossing (ISC). Organic–inorganic host matrices require moderately confined environments that can inhibit vibrational relaxation without entirely restricting configurational transitions. For radical-mediated systems, the photoinduced electron transfer (PIET) process must be finely tuned to generate and stabilize transient radicals while preventing undesired recombination under ambient conditions. These criteria require the concerted control of excited-state dynamics, spatial rigidity, and isomerization kinetics.

Each design strategy presents distinct advantages and limitations. Metal–ligand cooperative systems provide efficient ISC pathways and long-lived RTP but often suffer from poor processability and high synthesis costs. Host–guest and polymeric approaches offer greater structural tunability and flexibility but face difficulties in achieving long-term stability across operating cycles and environmental fluctuations. Radical-mediated systems enable dynamic, high-contrast luminescence switching, yet are constrained by the instability and poor reversibility of organic radicals. Moreover, most current systems rely heavily on UV-light excitation, which limits their applicability in bio-integrated and wearable technologies.

To enable real-world applications such as information encryption, optical storage, anti-counterfeiting, and dynamic bioimaging, photochromic–RTP materials must exhibit visible-light activation, long phosphorescence lifetimes, low fatigue, and high switching contrast. Promising strategies include the integration of multiple photoresponsive pathways (e.g., Z/E isomerization, enol–keto tautomerization) and the construction of multicomponent composite architectures (e.g., layered films, nanoconfined cages). These approaches aim to balance configurational flexibility with triplet state stabilization.

Notably, the emergence of artificial intelligence (AI) has begun to reshape research paradigms in this field. Machine learning (ML) and high-throughput virtual screening algorithms can accelerate the discovery and optimization of new RTP systems by predicting complex, nonlinear relationships between molecular structure and excited-state behavior [133,134]. In metal–organic and MOF-based systems, AI can assist in energy-level alignment optimization to maximize switching contrast. In host–guest systems, AI-guided models can help quantify the trade-offs between topological rigidity, configurational freedom, and nonradiative decay suppression.

Furthermore, deep learning and graph neural networks (GNNs) can facilitate the identification of multifunctional photoresponsive scaffolds, expediting the discovery of new photochromic units (e.g., Z/E isomers, light-gated radicals) and compatible RTP platforms. AI-assisted molecular dynamics simulations also contribute to the elucidation of excited-state deactivation pathways, conformational energy barriers, and their influence on phosphorescence lifetimes, offering theoretical support for exciton-level modulation.

In summary, future progress in this field will hinge on the integration of rational molecular engineering, interdisciplinary strategies, and data-driven tools. Synergistic design frameworks that combine energy-level tuning, dynamic confinement, and intelligent screening are essential to realize programmable, efficient, and durable photochromic–phosphorescent materials for next-generation optoelectronic and information technologies.

## 5. Summary and Outlook

This review summarizes the design principles and recent advances in light-responsive organic room-temperature phosphorescent (RTP) materials, outlining three general strategies: 1. Integration of photoisomerizable organic units into metal-coordination frameworks, complexes, or MOFs, enabling the coupling and regulation of photochromism and phosphorescence through metal center–ligand synergistic interactions. 2. Host–guest assembly combined with local rigidity design, where molecular motions are restricted while leaving configurational windows for isomerization. 3. In situ generation and stabilization of organic radicals via photoinduced electron transfer (PIET), constructing radical-based photochromic–RTP coupled systems.

Representative systems including isomerizable molecules, metal–organic complexes, organic–inorganic hybrids, purely organic radicals and COFs are discussed. Furthermore, their potential applications in reversible information encryption, bioimaging, and upconversion light-gated switching are highlighted.

Despite significant progress in developing light-responsive RTP across various materials, several critical challenges remain:

1. In metal center–ligand cooperative systems, precise energy-level matching between the isomerizable unit (pre-/post-isomerization) and the phosphorescent metal emitter is required to achieve high-contrast, low-loss RTP switching.
2. There is a need to expand the types of photochromic molecules beyond traditional ring-closing/opening species, introducing Z/E isomerization, enol–keto tautomerization, and photo-dimerization/de-dimerization to enrich photoregulation pathways and enhance photostability and fatigue resistance.
3. For the host–guest + local rigidity strategy, a quantitative understanding of the optimal balance between configurational freedom required for photochromism and rigidity required to suppress vibrational deactivation of RTP is essential. This demands in situ characterization and computational modeling to derive designable structural parameters.
4. In radical-mediated photochromic–RTP systems, radicals tend to spontaneously recombine in the dark, leading to poor color retention. Therefore, strategies such as steric shielding, ion pairing engineering, nanoscale confinement, or supramolecular encapsulation are required to improve radical lifetime and environmental stability while maintaining reversibility and cycling endurance.
5. From an application perspective, the materials should evolve toward visible-light activation, high quantum efficiency, long lifetime, and low fatigue, with extended applicability to quantum information storage, wearable optoelectronics, and intelligent sensing.
6. In the future, with the assistance of artificial intelligence (AI), the development of photochromic–RTP dual-functional materials is expected to shift from empirical exploration to a synergistic design paradigm driven by data and mechanistic understanding.

This transition will not only facilitate breakthroughs in visible-light activation, high quantum efficiency, long afterglow lifetime, and fatigue resistance, but also accelerate the practical deployment of these materials in quantum information storage, flexible wearable optoelectronics, and intelligent sensing and encoding systems.

Overall, by focusing on multidimensional coordination of energy-level alignment, structural confinement, and excited-state regulation—coupled with AI-powered molecular design and performance prediction strategies—new opportunities will emerge for constructing highly stable, high-contrast, and visible-light-responsive photo-triggered RTP systems, driving their real-world application and scalability in areas such as information storage, anti-counterfeiting encryption, biomedical imaging, and flexible optoelectronic devices.

## Data Availability

The data that support the findings of this study are available from the authors upon reasonable request.
